# Wearable Electrocardiography for Physical Activity Monitoring: Definition of Validation Protocol and Automatic Classification

**DOI:** 10.3390/bios13020154

**Published:** 2023-01-18

**Authors:** Gloria Cosoli, Luca Antognoli, Lorenzo Scalise

**Affiliations:** Department of Industrial Engineering and Mathematical Sciences, Università Politecnica delle Marche, v. Brecce Bianche snc, 60131 Ancona, Italy

**Keywords:** wearable sensors, validation protocol, electrocardiographic signal, heart rate, measurement accuracy, measurement uncertainty, machine learning, deep learning

## Abstract

Wearable devices are rapidly spreading thanks to multiple advantages. Their use is expanding in several fields, from medicine to personal assessment and sport applications. At present, more and more wearable devices acquire an electrocardiographic (ECG) signal (in correspondence to the wrist), providing potentially useful information from a diagnostic point of view, particularly in sport medicine and in rehabilitation fields. They are remarkably relevant, being perceived as a common watch and, hence, considered neither intrusive nor a cause of the so-called “white coat effect”. Their validation and metrological characterization are fundamental; hence, this work aims at defining a validation protocol tested on a commercial smartwatch (Samsung Galaxy Watch3, Samsung Electronics Italia S.p.A., Milan, Italy) with respect to a gold standard device (Zephyr BioHarness 3.0, Zephyr Technology Corporation, Annapolis, MD, USA, accuracy of ±1 bpm), reporting results on 30 subjects. The metrological performance is provided, supporting final users to properly interpret the results. Moreover, machine learning and deep learning models are used to discriminate between resting and activity-related ECG signals. The results confirm the possibility of using heart rate data from wearable sensors for activity identification (best results obtained by Random Forest, with accuracy of 0.81, recall of 0.80, and precision of 0.81, even using ECG signals of limited duration, i.e., 30 s). Moreover, the effectiveness of the proposed validation protocol to evaluate measurement accuracy and precision in a wide measurement range is verified. A bias of −1 bpm and an experimental standard deviation of 11 bpm (corresponding to an experimental standard deviation of the mean of ≈0 bpm) were found for the Samsung Galaxy Watch3, indicating a good performance from a metrological point of view.

## 1. Introduction

Wearable devices are continuously spreading, thanks to their multiple advantages. Indeed, they are user-friendly, minimally intrusive/invasive, and they can be easily adapted to different tasks and users’ needs [1], creating a plethora of relevant applications. Their use is very often combined with artificial intelligence (AI) algorithms and machine learning (ML) classifiers, for both prediction and regression purposes [2]. The application fields are the most various, from sleep stage classification [3] to automated diagnoses [4], as well as thermal comfort assessment [5], sport applications (e.g., rugby [6], football [7], swimming [8], and tennis [9]), rehabilitation, and management of patients with neurological disorders [10,11,12]. In fact, wearable sensors allow the assessment of a plethora of relevant physiological signals, like heart rate (HR) and its variability (HRV) [13,14] obtained via an ECG at wrist [15], the saturation of blood oxygen (SpO_2_) [16], and energy expenditure [17]. Also, diverse quantities can be indirectly estimated through dedicated procedures, like blood pressure [18,19] or breathing rate [20,21]. Such devices are extremely powerful in the context of remote monitoring and in recent years, there has been a continuously growing request for wireless wearables for diagnostic purposes, thanks to the development of AI technologies. Certainly, data quality is fundamental to obtain robust models: Dong [22] evaluated data leakage in ML algorithms ingesting data from sports wearable sensors, considering variables coupling through a Bayesian approach. The importance of data quality was stressed also by Kunze et al. [23], who evaluated the potential benefits of AI techniques in the orthopedic field, in the perspective of a real transformation of clinical practice towards more automated procedures (but the measurement results must be adequately accurate). Similarly, Xu et al. [24] highlighted the importance of identifying “free-of-noise” data to be ingested by ML algorithms for mobile health (mHealth) applications, in particular in the context of electrocardiographic (ECG) and respiratory signals.

It is worthy to note that ECG signals are mostly exploited to identify cardiovascular issues. Convolutional neural network (CNN) results tend to be the most utilized method (exploited in 52% of the analysed papers); gated recurrent unit (GRU) and long short-term memory (LSTM) are also very common for the identification of cardiac arrhythmias and abnormal heartbeats [25,26]. On the other hand, activities classification is commonly based on accelerometer and gyroscope data [27,28,29]. However, ECG-related features are also correlated to activity level, since the HR is expected to be higher during (and immediately after) a physical exertion [30]. The discrimination between rest and activity conditions can be particularly useful in the context of rehabilitation, where personalized therapeutic indications can be provided according to the activity level achieved by the patient at a precise moment. Furthermore, such distinction can be significant to evaluate the patient’s state in pathologies such as depression and other mood disorders, where activity represents an important discriminant [31]. This distinction made by a wrist-worn sensor is also more significant, since a smartwatch is not perceived as intrusive (not even by ageing people or subjects with mental disorders), thus it can provide results while avoiding the so-called “white coat effect”. On the other hand, literature studies focus more on the classifiers’ performance than on the metrological performance of the used sensors, leaving these aspects scarcely investigated, even if their relevance is very high, especially in health-related applications. It is worthy to remind the reader that the metrological performance can be intended as the ensemble of metrological characteristics describing the performance of a sensor from a metrological point of view, e.g., accuracy, precision, and confidence interval. In fact, the expanding medical applications of wearable sensors result in the need for validating the measurement signals. Only in this way can the gathered data be properly interpreted; moreover, it is possible to evaluate if the measurement accuracy is suitable for a specific application. Indeed, a proper metrological performance is required in such fields and the validity of wearable sensors must be demonstrated considering the needs of the target application. However, despite the rapidly growing use of wearable sensors, manufacturers/researchers rarely perform a rigorous metrological characterization. As a consequence, the measurement accuracy and precision data related to this type of measurement device are generally incomplete (or even missing). Moreover, to the best of the authors’ knowledge, at present, no test protocols have been defined and widely adopted to validate wearable devices from a metrological point of view. Each study is performed according to its own experimental procedure and the results are expressed with diverse metrics, resulting in data that are scarcely comparable to what is available in literature. Even when manufacturers provide information on measurement accuracy, the evaluation procedure remains unknown, rendering the evaluation non-replicable.

The authors would like to propose a meticulous method to characterize wearable sensors from a metrological point of view, providing rigorous evaluation metrics for the correct interpretation of the measurement results. Hence, the main aim of this study is to present a rigorous test protocol for the metrological characterization of wireless wearable devices for ECG monitoring and the assessment of HR. The metrological performance is evaluated on a commercial smartwatch in comparison to a reference device in terms of measurement accuracy and precision, linear correlation with a reference device, and statistical confidence of the measurement. Moreover, the authors used the ECG data collected from the wrist-worn wearable sensor as input for a few ML classifiers, which have been tested to distinguish among resting and activity-related signals (without using standard activity-related data, as those from accelerometers and gyroscope). Finally, the authors also tested an LSTM model for the same classification purposes, using raw ECG time-series segments.

The paper is organized as follows: Section 2 describes the study methodology (test protocol definition, acquisition devices and data processing until ML-based and LSTM-based classification) in detail; Section 3 reports the results; in Section 4, the authors provide discussion; finally, in Section 5 there are conclusions, along with some possible future developments of the study.

## 2. Materials and Methods

Healthy individuals were recruited as a test population and the experimental campaign was conducted at Università Politecnica delle Marche, Italy. All the subjects were informed of the study modalities and objectives and signed an informed consent module before participating in the study. The tests were performed in accordance with the WMA Declaration of Helsinki [32] and the data were managed according to the General Data Protection Regulation (GDPR). The Research Ethics Committee of Università Politecnica delle Marche approved the study, declaring it compliant with its Research Integrity Code.

**Figure 1:**
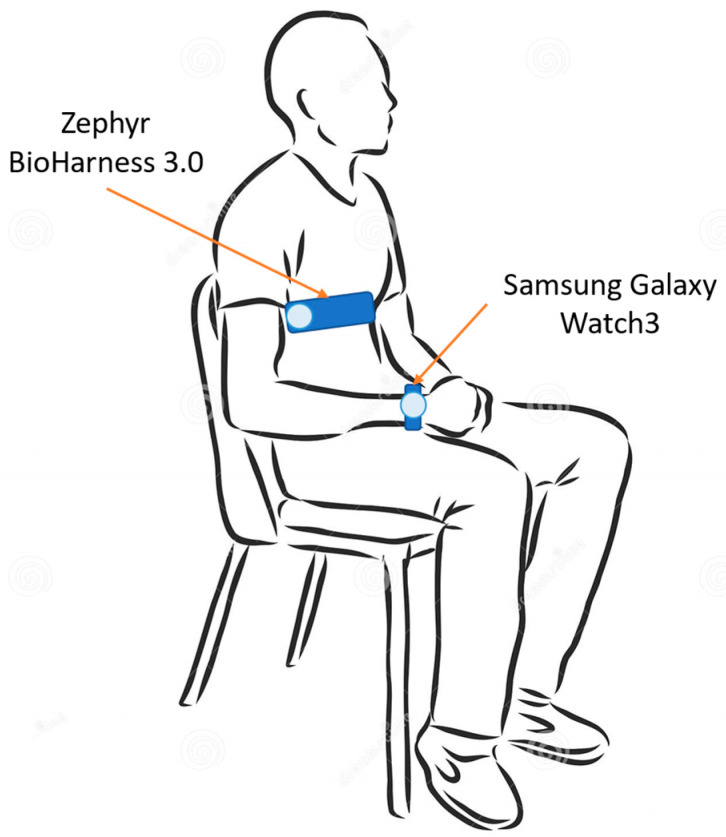
Measurement setup—recording phase is performed with the subject seated and still, both at rest and after physical activity.

Each subject was made wear the two acquisition devices. In particular, the test device (i.e., Samsung galaxy Watch3) was worn on the left wrist, whereas the reference instrument for ECG acquisition (i.e., Zephyr BioHarness 3.0) was worn on the chest. Both of the devices will be described in detail in the next section. The electrocardiographic signal was acquired by both the devices. The measurement setup is illustrated in Figure 1.

**Figure 2:**
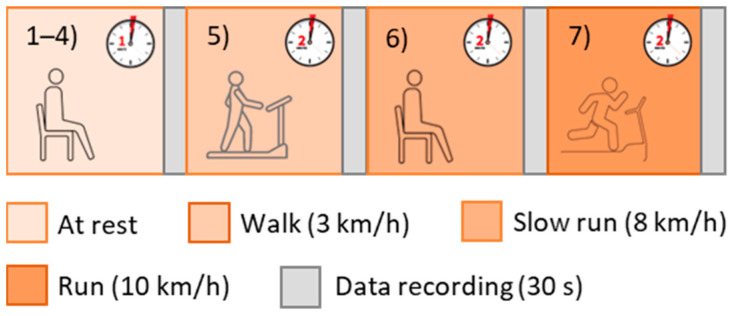
Test protocol.

Initially, the subjects were asked to stay at rest for 1 min in a seated position, then the recordings were started. In particular, each volunteer taking part in the study performed 7 tests and the recordings (with a 30-s duration) began just after each of them (Figure 2):

1. 4 tests in a resting condition;
2. 1 test after walking on a treadmill for 2 min at 3 km/h (with 0-slope);
3. 1 test after walking on a treadmill for 2 min at 8 km/h (with 0-slope);
4. 1 test after walking on a treadmill for 2 min at 10 km/h (with 0-slope).

It is worthy to underline that the subjects were asked to avoid speaking and remain as still as possible for the whole test duration, to minimize movement artefacts. Physical activity was considered to increase HR, but all the acquisitions were performed on the subjects seated at rest. In this way, bad quality data as a consequence of movements (particularly relevant if photoplethysmography-based devices should be validated) was avoided. The HR increase was necessary to widen the measurement range and evaluate the agreement between the test and reference devices in a broad measurement interval.

### 2.1. Acquisition Devices

**Figure 3:**
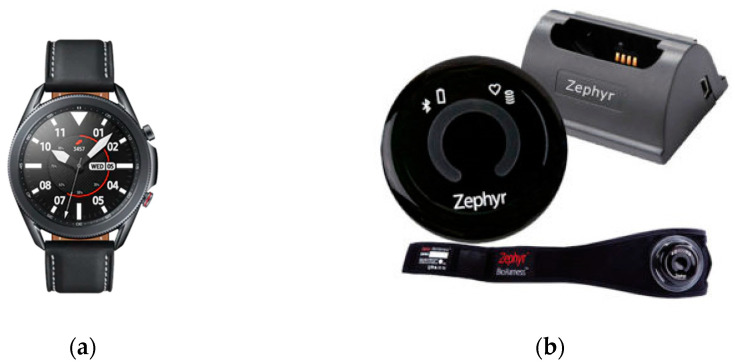
Acquisition devices: (**a**) test device: Samsung Galaxy Watch3 ECG [33]; (**b**) reference device: BioHarness 3.0 [34].

The test protocol proposed for the validation of wireless electrocardiographic monitors was applied to a commercial smartwatch (Samsung Galaxy Watch3, Figure 3a), which was evaluated in comparison to a gold standard system (Zephyr BioHarness 3.0, Figure 3b).

The Samsung Galaxy Watch3 is a smartwatch with advanced health technology; it is capable of providing not only standard HR (and related variability) measurement but can also monitor blood pressure (after an initial subject-dependent calibration), blood oxygen, and advanced running metrics (e.g., steps asymmetry and contact time). It is dust-tight and water-resistant up to 5 ATM (IP68 rating). Moreover, it can record the ECG waveform thanks to its embedded electrodes; the signal is displayed in real-time and recorded for subsequent evaluations.

The Zephyr BioHarness 3.0 [35] is a professional cardiac belt able to record an ECG signal, as well as a respiration signal and accelerometric data. It is water-resistant up to 1 m (IP55 rating). Its electrodes should be moistened with water before use to enhance electrical conductivity and, hence, signal quality. It has a declared accuracy of ±1 bpm for the HR measurement, in a measurement range of 0–140 bpm. The sampling frequency is equal to 250 Hz.

### 2.2. Data Analysis for Metrological Characterization

The gathered ECG signals were processed in a MATLAB^®^ (1994–2023 The MathWorks, Inc.) environment to extract the related tachogram, that is the inter-beat intervals (IBIs) succession. After having detrended the recorded ECG signals (removing the best straight-fit line), the Pan-Tompkins algorithm was exploited to detect R peaks in the signals coming from both the test and reference devices. Then, the test and reference tachograms were computed as the successions of IBIs.

In order to evaluate the metrological performance of the test device, the measurement differences between test and reference signals (in terms of HR) were considered and the comparison was made beat-to-beat. At first, the signals were synchronized thanks to the timestamps saved by the two acquisition devices; the outliers were removed. It is worthy to note that data were considered as outliers when they were outside of the 99.7% confidence interval of the experimental distribution (coverage factor k = 3).

The measurement differences were analyzed according to standard statistical methods, namely:

- Distribution of deviations: it is expected that measurement differences assume a Gaussian-like distribution. Both mean (µ) and standard deviation (σ) values of such experimental measurement differences are computed, being associated with measurement accuracy and precision, respectively;
- Analysis of agreement, which is conducted through the Bland–Altman plot [36]. This graph plots the measurement differences with respect to the expected measurement value (i.e., the mean value between test and reference measurements). In this way, it is possible to derive the confidence interval at 95% (CI95%) of the levels of agreement (meaningful of the statistical confidence of the measurement), as well as the bias (the mean value of the measurement differences—substantially coincident with the µ value obtained from the distribution of deviations);
- Correlation between the test and reference signals and computation of the related Pearson’s correlation coefficient (ρ). Specifically, the strength of the linear correlation is evaluated as strong for ρ > 0.7, moderate for 0.3 < ρ < 0.7, and low for ρ < 0.3 [37].

### 2.3. ML-Based Classification

Different supervised ML algorithms, suitable for both classification and regression purposes, were exploited to classify the resting/(post-)activity conditions of the tested subject, namely:

- Support vector machine (SVM) [38]: it is based on statistical learning frameworks. It sets a model assigning an item to a category in a non-probabilistic classification setting, maximizing the gap between different categories;
- Random forest (RF) [39]: it realizes different decision trees in the training phase. It corrects overfitting issues and usually outperforms decision trees;
- Simple logistic (SL) [40]: it builds a linear logistic regression model based on the determination of the relationship between two variables, fitting a logistic curve to binary data;
- Decision table (DT) [41]: it can be used for a numeric prediction starting from decision trees. It is based on if–then rules and is more compact than decision trees;
- Naïve Bayes (NB) [42]: it assumes that a feature value does not depend on the others and that there are no correlations among them. It uses the method of maximum likelihood, with or without Bayesian probability methods.

As classification labels, two items were considered: rest conditions and (post-)activity. It should be considered that all the recordings were performed at rest or just after the execution of physical exertion. Hence, the effect of activity on the physiological signal is related to the recovery time of the subject and depends on the training level of the tested subject, among other factors. The data were divided into two subsets for training and testing, with a ratio of 7:3. The input variables were ECG features in time domain, namely:

- Mean value and standard deviation of HR;
- The root mean square of successive inter-beat intervals (RMSSD);
- The percentage of successive RR intervals (i.e., IBIs, intended as differences between two consequent R peaks) differing for a value greater than 50 ms (pNN50).

The database was composed of 210 trials acquired on the whole test population (30 subjects, 7 tests on each) through the smartwatch. The classification performance was evaluated through standard metrics [43], namely accuracy, recall, precision, F-measure, and area under ROC curve. The ML-based classification was performed within the WEKA toolbox from Waikato University [44]; a 10-fold cross-validation method was adopted and the reported metrics are the values averaged on the 10 iterations.

Moreover, the authors exploited an AI neural network to discriminate between the signals related to rest conditions and the signals acquired just after the execution of physical activity. In particular, an LSTM model was exploited; it is a type of recurrent neural network (RNN) suitable to deal with time series and to examine long-term dependencies [25]. It employs memory blocks (i.e., one or more memory cells) and not simple RNN units; input and output are considered as multiplicative gates, controlling the information flow to memory cells. In particular, the LSTM model used is formed by a 1 × 5 array of layers, specifically:

- Input layer;
- Bidirectional-LSTM (BiLSTM) with 100 hidden units;
- 9 fully connected layer;
- Softmax;
- Output layer.

The model was implemented in a MATLAB^®^ environment through the Deep Learning Toolbox. The database was formed by the raw time series data extracted from the electrocardiographic signals acquired through the test device (smartwatch). In particular, the database was constituted by a total of 6680 segments, with a duration of 0.4 s each, identified around the R-peak (the R peak is located at 0.1 s of the segment). The dataset was divided into two subsets of training and testing, with a ratio of 7:3; a 10-fold cross-validation method was exploited.

## 3. Results

The test population was formed by 30 healthy subjects (11 males, 19 females), with an age of 22 ± 3 years and a body mass index (BMI) of (22.5 ± 2.3) kg/m^2^ (the data are reported as mean ± standard deviation). The related demographics are reported in Table 1.

The results related to the metrological characterization of the test device and the activity level classification through ML algorithms and LSTM model are reported in the following sections.

### 3.1. Measurement Accuracy and Precision

**Figure 4:**
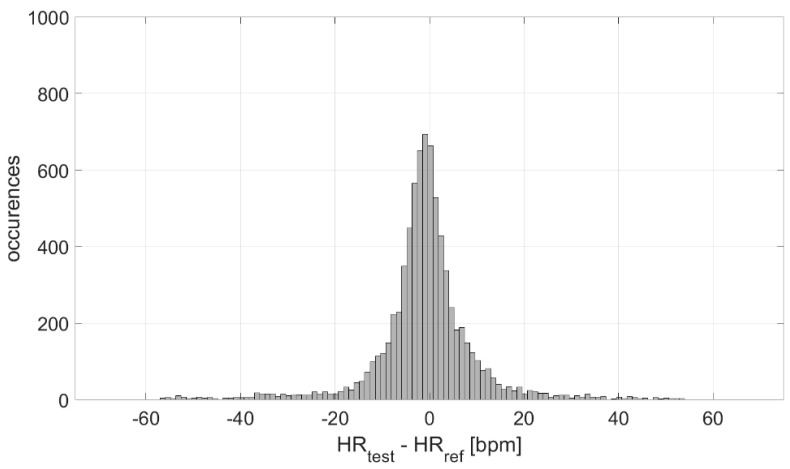
Distribution of measurement differences.

The measurement differences in terms of HR measured by the test and reference devices were analyzed, at first, in terms of distribution. The result is graphed in Figure 4; a Gaussian-like distribution is obtained, with a mean value of −1 bpm and an experimental standard deviation value equal to 11 bpm. Considering the number of occurrences, the experimental standard deviation of the mean value is ≈0 bpm, indicating a high accuracy in the estimation of the mean measurement difference (−1 bpm).

**Figure 5:**
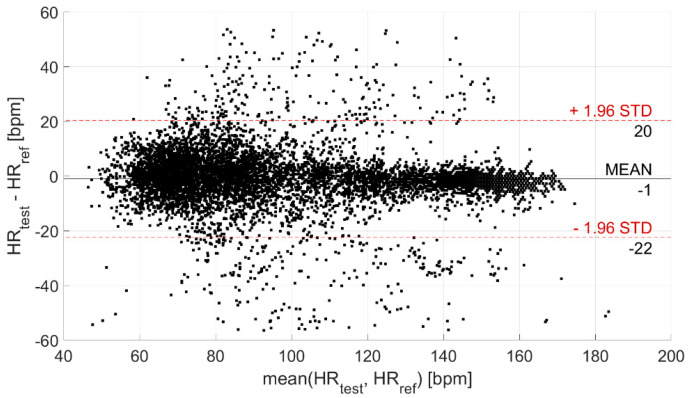
Bland–Altman plot. The black horizontal line indicates the mean measurement difference (i.e., bias), whereas the two red horizontal lines indicate the limits of the confidence interval at 95%.

Then, the agreement between the test and reference devices was evaluated through the Bland–Altman plot, illustrated in Figure 5. In this way, the CI95% of the levels of agreement was derived; it is equal to (−22; 20) bpm (coverage factor k = 2) and is indicative of the statistical confidence of the measurement.

**Figure 6:**
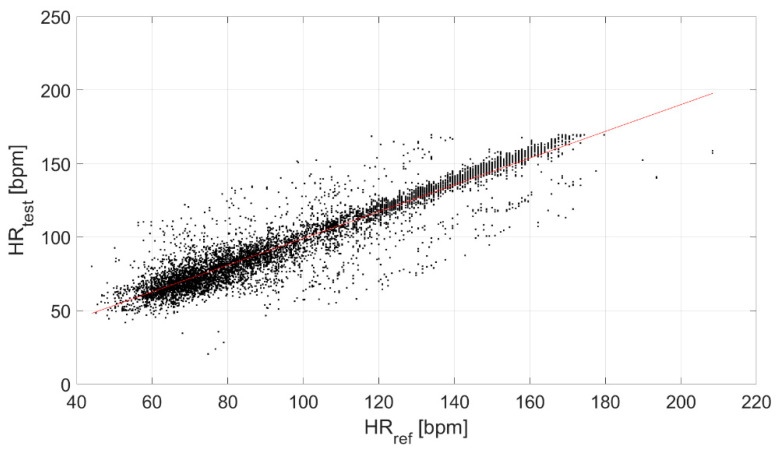
Correlation between the test and reference devices.

Finally, the correlation between the test and reference devices was evaluated (Figure 6), obtaining a ρ equal to 0.94.

### 3.2. Physical Activity Level Classification

The performance of the tested ML classifiers and LSTM model in the discrimination among rest and (post-)activity conditions is reported in Table 2 in terms of accuracy, recall, precision, F-measure, and area under ROC curve, along with the number of true positives and negatives (i.e., TP correctly classified (post-)activity-related signals, and TN correctly classified rest-related signals, respectively). and the number of false positives and negatives (i.e., FP incorrectly classified (post-)activity-related signals, and FN incorrectly classified rest-related signals, respectively). It is worthy to note that the weighted averages on the identification of the two conditions (i.e., rest and (post-)activity) are reported for the performance metrics.

## 4. Discussion

In this section, the obtained results are discussed.

### 4.1. Measurement Accuracy and Precision

The distribution of the measurement differences between the test and reference sensors is Gaussian (Figure 4). Such differences are clenched around −1 bpm, indicating a high measurement accuracy (also suitable for medical purposes), with an associated experimental standard deviation of approximately 0 bpm (indicating a very good estimation of accuracy). However, the experimental standard deviation value of the distribution (characterizing the dispersion about the mean value, which is an index of the measurement precision) is equal to 11 bpm. This value is not suitable for clinical applications, which indicates the need to improve these sensors from a hardware point of view.

The analysis of the agreement between the test and reference devices, performed through the Bland–Altman plot (Figure 5), provided a CI95% of (−22; 20) bpm (coverage factor k = 2) for the levels of agreement, related to the statistical confidence of the measurement. Despite a quite large confidence interval, the measurement of HR is very accurate. Indeed, the mean difference with respect to the reference device is equal to −1 bpm (as already observed through the analysis of measurement differences distribution).

Finally, a strong linear correlation of the test device with the reference sensor was found (ρ equal to 0.94, Figure 6).

Summarizing these findings, it can be stated that the test device appears to be very accurate, even if the statistical confidence for the levels of agreement in the HR assessment is limited. Indeed, the device provides a very low mean difference (i.e., −1 bpm) with an experimental standard deviation of 11 bpm. From a clinical point of view, these figures are relevant, since the sensors are accurate, but not precise enough. Hence, the target application should always be considered to properly evaluate the desired metrological performance. This is the reason why wearable devices are not yet diffused in clinical practices, especially for diagnostic purposes. Regardless, their role in the remote monitoring of patients (e.g., ageing people living at home [45]) can be relevant, since changes in the observed trend can be detected relatively easily. Research is very active in this field [46,47,48], but further steps are required to achieve suitable accuracy and precision before a wide adoption of wearables in a medical environment for diagnostic purposes.

Considering the Samsung Galaxy Watch3, to the best of the authors’ knowledge, at present, there are no previous studies characterizing its metrological performance, and the manufacturer does not provide this information. Furthermore, it is worthy to note that terminology is often confused and what is reported as precision is indeed measurement accuracy (especially in the wearables’ user manuals). The results surely depend on the measurement range, as well as on the hardware and embedded software (since a lot of sensors filter the acquired signals before providing results, e.g., Empatica E4 [49]). Indeed, in a previous study the authors tested multiple devices for HR measurement, evidencing standard deviations of measurement differences at rest equal to 4 bpm for different smartwatch models (i.e., Empatica E4, Polar Vantage V2, and Garmin Venu Sq) [50]. On the other hand, Sequeira et al. [51] underlined the inaccuracy of common wearable devices (i.e., Apple, Fitbit, Garmin, and Polar) in HR measurement during supraventricular tachycardia. Hence, it is plausible that smartwatches are accurate in rest conditions, but their performance significantly decreases when movements or arrhythmias are present.

### 4.2. Physical Activity Level Classification

In Table 2**,** it can be noticed that RF is the best performing classifier, with an accuracy equal to 0.81. All the metrics confirm this result; in fact, values of 0.80, 0.81, 0.80, and 0.84 were obtained for Recall, Precision, F-measure, and ROC area, respectively. Also, the classification performance of SVM and RF are good, with an accuracy of 0.78 and 0.76, respectively. The worst performance was obtained for DT classifier, reporting an accuracy of 0.66.

Moreover, the set AI neural network proved to be suitable to discriminate between signals related to resting periods and those concerning activities. In fact, the trained LSTM model provided an accuracy of 0.79, with a recall of 0.72 and a precision of 0.73 (Table 2). It is possible to note that the performance is similar to the values obtained for the tested ML classifiers (in particular, accuracy is similar to that obtained for RF, which, however, reported better results for the other metrics).

The classification performance would be better if a larger dataset was used, hence the limited sample size should be considered as a limitation of the present study. Another limitation is the scarce heterogeneity of the test population demographics. Moreover, it is worthy to underline that the rest/(post-)activity classification has been made only considering the ECG signal and the derived HR. Conversely, in literature, the activity classification often relies on movement-related sensors (e.g., accelerometers [52]) and sometimes includes multimodal parameters [53], achieving better classification performance values. For example, Bijalwan et al. [54] tested four deep learning models, finding accuracies of 0.58, 0.84, 0.86, and 0.90 for deep neural network, bidirectional-long short-term memory (BLSTM), CNN, and CNN-LSTM, respectively. Hence, in the future, it would be interesting to expand the input to the AI models considering different signals acquired through wearable devices (e.g., data from accelerometer, used to discriminate between activity and rest conditions, but also to correct eventual motion artifact in cardiac-related signals).

Also, in this case, it must be underlined that the performance metrics obtained are not adequate for a clinical application, but only for monitoring purposes. Again, this can provide a relevant support for remote monitoring of the elderly or in sport applications, but their role in medicine will be very limited until further steps are taken. For example, the classification models should be developed based on very large samples, properly including physiological variability and, hence, more easily generalizable. For this reason, the present experimentation can be considered as a pilot study that needs additional developments to achieve broader results.

## 5. Conclusions

In this paper, the authors have defined a test protocol to characterize wireless devices for electrocardiographic signals acquisition and HR monitoring from a metrological point of view. A wide picture of the device metrological performance is provided, highlighting relevant features, such as measurement accuracy and precision. In particular, the protocol foresees the execution of physical activities intended to increase the subject’s HR, to widen the considered measurement range and broaden the metrological evaluation of the test device. In this way, the performance can be discussed in a broader measurement range to evaluate possible variations with HR values and, hence, activity level. This is fundamental for the validation of a wearable sensor, whose measurement results should always be provided with the related measurement uncertainty. Indeed, the measurement accuracy of a sensor should be in line with the target application requirements.

Moreover, the authors evaluated the classification performance of a few ML algorithms and of an AI model to distinguish among rest and (post-)activity conditions. This can be useful, for example, to provide the user some tips during training (e.g., sport applications [55,56]) or rehabilitation (e.g., ambient assisted-living field [57]) phase, hence optimizing the benefits of the physical activity itself. On the other hand, such an indication could be employed in Industry 4.0 context [58], were physiological data, combined with data coming from work processes can help in optimizing the worker’s conditions and, thus, productivity. Additionally, the discrimination of rest/activity based on ECG data coming from a smartwatch could also be beneficial in the management of mood disorders [59,60], where the activity level is a pivotal marker of the patient’s state. Also, ageing patients, who may be affected by Alzheimer’s or Parkinson disease, can benefit from such a tool, since activity also characterizes sleep and circadian rhythms, providing meaningful information on the patient’s condition [61,62].

The proposed test protocol has been evaluated on a commercial wrist-worn device (Samsung Galaxy Watch3), which has been tested on 30 healthy subjects. The results obtained through the analysis of measurement differences between the test and reference devices show a mean value of measurement differences (linked to measurement accuracy) of -1 bpm, with an experimental standard deviation of 11 bpm, indicating a high variability of the measurement differences. Hence, the tested wearable sensor can be considered accurate even if not very precise, presenting a 95% statistical confidence of (−22; 20) bpm (coverage factor k = 2) in relation to the levels of agreement.

For what concerns ML-based classification, the RF classifier proved to be the best performing one, with a classification accuracy of 0.81; on the other hand, the tested LSTM model provided an accuracy of 0.79, comparable to the ML algorithms. The classification performance is acceptable, even if results are often lower with respect to the studies available in literature (reaching accuracies even beyond 0.90). However, it is worthy to note that only a few features extracted from the electrocardiographic have been exploited in this study and other signals have not been considered. Moreover, all the acquisitions were made while the subject was in a static condition (at rest or just after having performed a physical task), to prioritize the evaluation of the signal quality, minimizing movement artefacts.

The proposed experimental protocol has been tested for the validation of wearable sensors in a wide measurement range, even if on a limited population, and no major concerns have to be underlined. Hence, the present work can be considered a good pilot study, which encourages widening the test protocol application to different sensors, as well as measuring diverse physiological parameters (always employing a suitable reference). It is worthy to underline that the test population characteristics can undoubtedly influence the results; including as much physiological variability as possible contributes to improvement of the results in terms of robustness and applicability in different contexts.

In the future, the application of the test protocol will be extended to further parameters than HR values: the diverse ECG peaks location, the waveform frequency content, and the R-peak amplitude. These features could also be useful for classification, where it would be interesting to widen the test population and, hence, use a larger dataset to train the models. In fact, these types of algorithms learn better (and, hence, perform better) if a larger input variability is provided. In this regard, the age and BMI ranges of the test population should also be widened to include more heterogeneity in the tested sample. This way, diverse levels of activities could also be classified, going beyond the scope of a binary classification.

## Figures and Tables

**Table 1:** Demographics of the test population (age, weight, height, and BMI) reported in terms of mean, standard deviation of population, and confidence interval for the mean value (significance level: 0.05).

	Mean	Standard Deviation	Confidence Interval at 95%
Age	22 years	3 years	(21, 23) years
Weight	64 kg	9 kg	(61, 67) kg
Height	1.69 m	0.07 m	(1.67, 1.71) m
BMI	22.5 kg/m^2^	2.3 kg/m^2^	(21.6, 23.3) kg/m^2^

**Table 2:** ML classifiers and LSTM model performance in the classification of resting/activities-related signals. TP are the true positives (i.e., correctly classified (post-)activity-related signals), TN the true negatives (i.e., correctly classified rest-related signals), FP the false positives (i.e., incorrectly classified (post-)activity-related signals), and FN the false negatives (i.e., incorrectly classified rest-related signals).

Model	Accuracy	Recall	Precision	F-Measure	ROC Area	TP	TN	FP	FN
SVM	0.78	0.78	0.83	0.78	0.80	26	23	12	1
**RF**	**0.81**	**0.80**	**0.81**	**0.80**	**0.84**	**24**	**26**	**9**	**3**
SL	0.70	0.70	0.70	0.70	0.79	19	25	10	8
DT	0.66	0.66	0.67	0.64	0.80	11	30	5	16
NB	0.76	0.76	0.82	0.76	0.76	27	21	14	1
LSTM	0.79	0.62	0.73	0.67	0.84	1180	442	276	166

## Data Availability

Data can be obtained from the corresponding authors upon request.

## References

[1] Vijayan V., Connolly J.P., Condell J., McKelvey N., Gardiner P. (2021). Review of Wearable Devices and Data Collection Considerations for Connected Health. Sensors.

[2] Zhang S., Li Y., Zhang S., Shahabi F., Xia S., Deng Y., Alshurafa N. (2022). Deep Learning in Human Activity Recognition with Wearable Sensors: A Review on Advances. Sensors.

[3] Chen P.-W., O’Brien M.K., Horin A.P., Koch L.L.M., Lee J.Y., Xu S., Zee P.C., Arora V.M., Jayaraman A. (2022). Sleep Monitoring during Acute Stroke Rehabilitation: Toward Automated Measurement Using Multimodal Wireless Sensors. Sensors.

[4] Santucci F., Presti D.L., Massaroni C., Schena E., Setola R. (2022). Precordial Vibrations: A Review of Wearable Systems, Signal Processing Techniques, and Main Applications. Sensors.

[5] Cosoli G., Mansi S.A., Arnesano M. Combined use of wearable devices and Machine Learning for the measurement of thermal sensation in indoor environments. Proceedings of the 2022 IEEE International Workshop on Metrology for Living Environment (MetroLivEn).

[6] Carey L., Stanwell P., Terry D.P., McIntosh A.S., Caswell S.V., Iverson G.L., Gardner A.J. (2019). Verifying Head Impacts Recorded by a Wearable Sensor using Video Footage in Rugby League: A Preliminary Study. Sports Med. Open.

[7] Di Paolo S., Zaffagnini S., Pizza N., Grassi A., Bragonzoni L. (2021). Poor Motor Coordination Elicits Altered Lower Limb Biomechanics in Young Football (Soccer) Players: Implications for Injury Prevention through Wearable Sensors. Sensors.

[8] Cosoli G., Antognoli L., Veroli V., Scalise L. (2022). Accuracy and Precision of Wearable Devices for Real-Time Monitoring of Swimming Athletes. Sensors.

[9] Wu M., Wang R., Hu Y., Fan M., Wang Y., Li Y., Wu S. (2021). Invisible experience to real-time assessment in elite tennis athlete training: Sport-specific movement classification based on wearable MEMS sensor data. Proc. Inst. Mech. Eng. Part P J. Sports Eng. Technol..

[10] Adams J.L., Dinesh K., Snyder C.W., Xiong M., Tarolli C.G., Sharma S., Dorsey E.R., Sharma G. (2021). A real-world study of wearable sensors in Parkinson’s disease. NPJ Parkinsons Dis..

[11] Mughal H., Javed A.R., Rizwan M., Almadhor A.S., Kryvinska N. (2022). Parkinson’s Disease Management via Wearable Sensors: A Systematic Review. IEEE Access.

[12] Wilson R., Vangala S., Elashoff D., Safari T., Smith B. (2021). Using Wearable Sensor Technology to Measure Motion Complexity in Infants at High Familial Risk for Autism Spectrum Disorder. Sensors.

[13] Hinde K., White G., Armstrong N. (2021). Wearable Devices Suitable for Monitoring Twenty Four Hour Heart Rate Variability in Military Populations. Sensors.

[14] Mohapatra P., Premkumar P.S., Sivaprakasam M. (2018). A Yellow–Orange Wavelength-Based Short-Term Heart Rate Variability Measurement Scheme for Wrist-Based Wearables. IEEE Trans. Instrum. Meas..

[15] Rahman M., Morshed B.I. Extraction of Respiration Rate from Wrist ECG Signals. Proceedings of the 2021 IEEE 12th Annual Ubiquitous Computing, Electronics & Mobile Communication Conference (UEMCON).

[16] Singh P., Kaur G., Kaur D. (2017). Infant Monitoring System Using Wearable Sensors Based on Blood Oxygen Saturation: A Review. Intelligent, Secure, and Dependable Systems in Distributed and Cloud Environments.

[17] Cvetkovic B., Milic R., Lustrek M. (2016). Estimating Energy Expenditure with Multiple Models Using Different Wearable Sensors. IEEE J. Biomed. Health Informatics.

[18] Arakawa T. (2018). Recent Research and Developing Trends of Wearable Sensors for Detecting Blood Pressure. Sensors.

[19] Poli A., Cosoli G., Iadarola G., Spinsante S., Scalise L. Feasibility of Blood Pressure Measurement through Wearable Devices: Analysis of Smartwatches Performance. Proceedings of the 2022 IEEE International Symposium on Medical Measurements and Applications (MeMeA).

[20] Prigent G., Aminian K., Rodrigues T., Vesin J.-M., Millet G.P., Falbriard M., Meyer F., Paraschiv-Ionescu A. (2021). Indirect Estimation of Breathing Rate from Heart Rate Monitoring System during Running. Sensors.

[21] Cosoli G., Antognoli L., Scalise L. Indirect Estimation of Breathing Rate through Wearable Devices. Proceedings of the 2022 IEEE International Symposium on Medical Measurements and Applications (MeMeA).

[22] Dong Q. (2022). Leakage Prediction in Machine Learning Models When Using Data from Sports Wearable Sensors. Comput. Intell. Neurosci..

[23] Kunze K.N., Orr M., Krebs V., Bhandari M., Piuzzi N.S. (2022). Potential benefits, unintended consequences, and future roles of artificial intelligence in orthopaedic surgery research. Bone Jt. Open.

[24] Xu H., Yan W., Lan K., Ma C., Wu D., Wu A., Yang Z., Wang J., Zang Y., Yan M. (2021). Assessing Electrocardiogram and Respiratory Signal Quality of a Wearable Device (SensEcho): Semisupervised Machine Learning-Based Validation Study. JMIR mHealth uHealth.

[25] Ebrahimi Z., Loni M., Daneshtalab M., Gharehbaghi A. (2020). A review on deep learning methods for ECG arrhythmia classification. Expert Syst. Appl. X.

[26] Rana A., Kim K.K. ECG Heartbeat Classification Using a Single Layer LSTM Model. Proceedings of the 2019 International SoC Design Conference (ISOCC).

[27] Mekruksavanich S., Jitpattanakul A. Smartwatch-based Human Activity Recognition Using Hybrid LSTM Network. Proceedings of the 2020 IEEE SENSORS.

[28] Amor J.D., James C.J. (2018). Validation of a Commercial Android Smartwatch as an Activity Monitoring Platform. IEEE J. Biomed. Health Informatics.

[29] Kheirkhahan M., Nair S., Davoudi A., Rashidi P., Wanigatunga A.A., Corbett D.B., Mendoza T., Manini T.M., Ranka S. (2019). A smartwatch-based framework for real-time and online assessment and mobility monitoring. J. Biomed. Informatics.

[30] Izmailova E.S., McLean I.L., Hather G., Merberg D., Homsy J., Cantor M., Volfson D., Bhatia G., Perakslis E.D., Benko C. (2019). Continuous Monitoring Using a Wearable Device Detects Activity-Induced Heart Rate Changes after Administration of Amphetamine. Clin. Transl. Sci..

[31] Moraes C., Cambras T., Diez-Noguera A., Schimitt R., Dantas G., Levandovski R., Hidalgo M.P. (2013). A new chronobiological approach to discriminate between acute and chronic depression using peripheral temperature, rest-activity, and light exposure parameters. BMC Psychiatry.

[32] WMA Declaration of Helsinki—Ethical Principles for Medical Research Involving Human Subjects—WMA—The World Medical Association. https://www.wma.net/policies-post/wma-declaration-of-helsinki-ethical-principles-for-medical-research-involving-human-subjects/.

[33] Samsung Galaxy Watch 3|Samsung UK. https://www.samsung.com/uk/watches/galaxy-watch/galaxy-watch3-45mm-mystic-black-lte-sm-r845fzkaeua/.

[34] Zephyr BioHarness BTLE ECHO Module (Gen 3)|HaB Direct. https://www.habdirect.co.uk/product/zephyr-bioharness-btle-echo-module-gen-3/.

[35] (2012). BioHarness 3.0 User Manual. www.zephyanywhere.com.

[36] Altman D.G., Bland J.M. (1983). Measurement in Medicine: The Analysis of Method Comparison Studies. J. R. Stat. Soc. Ser. D Stat..

[37] Akoglu H. (2018). User’s guide to correlation coefficients. Turk. J. Emerg. Med..

[38] Ifenthaler D., Widanapathirana C. (2014). Development and Validation of a Learning Analytics Framework: Two Case Studies Using Support Vector Machines. Technol. Knowl. Learn..

[39] Balyan A.K., Ahuja S., Lilhore U.K., Sharma S.K., Manoharan P., Algarni A.D., Elmannai H., Raahemifar K. (2022). A Hybrid Intrusion Detection Model Using EGA-PSO and Improved Random Forest Method. Sensors.

[40] Kirasich K., Smith T., Sadler B. (2018). Random Forest vs. Logistic Regression: Binary Classification for Heterogeneous Datasets. SMU Data Sci. Rev..

[41] Huysmans J., Dejaeger K., Mues C., Vanthienen J., Baesens B. (2011). An empirical evaluation of the comprehensibility of decision table, tree and rule based predictive models. Decis. Support Syst..

[42] Jiang L., Zhang L., Li C., Wu J. (2019). A Correlation-Based Feature Weighting Filter for Naive Bayes. IEEE Trans. Knowl. Data Eng..

[43] Cook D.J. Activity learning: Discovering, Recognizing, and Predicting Human Behavior from Sensor Data. https://www.wiley.com/en-gb/Activity+Learning%3A+Discovering%2C+Recognizing%2C+and+Predicting+Human+Behavior+from+Sensor+Data-p-9781118893760.

[44] Hall M., Frank E., Holmes G., Pfahringer B., Reutemann P., Witten I.H. (2009). The WEKA data mining software: An update. SIGKDD Explor. Newsl..

[45] Stavropoulos T.G., Papastergiou A., Mpaltadoros L., Nikolopoulos S., Kompatsiaris I. (2020). IoT Wearable Sensors and Devices in Elderly Care: A Literature Review. Sensors.

[46] Mamdiwar S.D., R A., Shakruwala Z., Chadha U., Srinivasan K., Chang C.-Y. (2021). Recent Advances on IoT-Assisted Wearable Sensor Systems for Healthcare Monitoring. Biosensors.

[47] Majumder S., Mondal T., Deen M.J. (2017). Wearable Sensors for Remote Health Monitoring. Sensors.

[48] Pillai S., Upadhyay A., Sayson D., Nguyen B.H., Tran S.D. (2022). Advances in Medical Wearable Biosensors: Design, Fabrication and Materials Strategies in Healthcare Monitoring. Molecules.

[49] E4 Wristband|Real-Time Physiological Signals|Wearable PPG, EDA, Temperature, Motion Sensors. https://www.empatica.com/en-eu/research/e4/.

[50] Cosoli G., Poli A., Antognoli L., Spinsante S., Scalise L. What is my heart rate right now? Comparing data from different devices. Proceedings of the 2022 IEEE International Instrumentation and Measurement Technology Conference (I2MTC).

[51] Sequeira N., D’Souza D., Angaran P., Aves T., Dorian P. (2020). Common wearable devices demonstrate variable accuracy in measuring heart rate during supraventricular tachycardia. Heart Rhythm..

[52] Erdaş Ç.B., Güney S. (2021). Human Activity Recognition by Using Different Deep Learning Approaches for Wearable Sensors. Neural Process. Lett..

[53] Cosoli G., Poli A., Scalise L., Spinsante S. (2021). Measurement of multimodal physiological signals for stimulation detection by wearable devices. Measurement.

[54] Bijalwan V., Semwal V.B., Gupta V. (2022). Wearable sensor-based pattern mining for human activity recognition: Deep learning approach. Ind. Robot. Int. J. Robot. Res. Appl..

[55] Bleser G., Steffen D., Reiss A., Weber M., Hendeby G., Fradet L. (2015). Personalized physical activity monitoring using wearable sensors. Smart Health.

[56] Seshadri D.R., Li R.T., Voos J.E., Rowbottom J.R., Alfes C.M., Zorman C.A., Drummond C.K. (2019). Wearable sensors for monitoring the internal and external workload of the athlete. NPJ Digit. Med..

[57] Ranieri C.M., MacLeod S., Dragone M., Vargas P.A., Romero R.A.F. (2021). Activity Recognition for Ambient Assisted Living with Videos, Inertial Units and Ambient Sensors. Sensors.

[58] Donati M., Olivelli M., Giovannini R., Fanucci L. RT-PROFASY: Enhancing the Well-being, Safety and Productivity of Workers by Exploiting Wearable Sensors and Artificial Intelligence. Proceedings of the 2022 IEEE International Workshop on Metrology for Industry 4.0 & IoT (MetroInd4.0&IoT).

[59] Victory A., Letkiewicz A., Cochran A.L. (2020). Digital solutions for shaping mood and behavior among individuals with mood disorders. Curr. Opin. Syst. Biol..

[60] Taj-Eldin M., Ryan C., O’Flynn B., Galvin P. (2018). A Review of Wearable Solutions for Physiological and Emotional Monitoring for Use by People with Autism Spectrum Disorder and Their Caregivers. Sensors.

[61] Buchman A.S., Dawe R.J., Leurgans S.E., Curran T.A., Truty T., Yu L., Barnes L.L., Hausdorff J.M., Bennett D.A. (2012). Different Combinations of Mobility Metrics Derived from a Wearable Sensor Are Associated with Distinct Health Outcomes in Older Adults. Journals Gerontol. Ser. A.

[62] Madrid-Navarro C.J., Sevilla F.E., Mínguez-Castellanos A., Campos M., Ruiz-Abellán F., Madrid J.A., Rol M.A. (2018). Multidimensional Circadian Monitoring by Wearable Biosensors in Parkinson’s Disease. Front. Neurol..

